# A machine learning approach for retinal images analysis as an objective screening method for children with autism spectrum disorder

**DOI:** 10.1016/j.eclinm.2020.100588

**Published:** 2020-11-05

**Authors:** Maria Lai, Jack Lee, Sally Chiu, Jessie Charm, Wing Yee So, Fung Ping Yuen, Chloe Kwok, Jasmine Tsoi, Yuqi Lin, Benny Zee

**Affiliations:** a Centre for Clinical Research and Biostatistics, Jockey Club School of Public Health and Primary Care, The Chinese University of Hong Kong, Hong Kong SAR; b Clinical Trials and Biostatistics Lab, CUHK Shenzhen Research Institute, Shenzhen, China; c The Hong Chi Association, Hong Kong SAR; d Sight Enhancement Center, Hong Kong SAR; e The Jockey Club Hong Chi School, Wan Chai, Hong Kong SAR; f The Hong Chi Morninghill School, Tuen Mun, Hong Kong SAR

**Keywords:** Autism spectrum disorder, Automatic retinal image analysis, Machine learning, screening tool, Risk assessment

## Abstract

**Background:**

Autism spectrum disorder (ASD) is characterised by many of features including problem in social interactions, different ways of learning, some children showing a keen interest in specific subjects, inclination to routines, challenges in typical communication, and particular ways of processing sensory information. Early intervention and suitable supports for these children may make a significant contribution to their development. However, considerable difficulties have been encountered in the screening and diagnosis of ASD. The literature has indicated that certain retinal features are significantly associated with ASD. In this study, we investigated the use of machine learning approaches on retinal images to further enhance the classification accuracy.

**Methods:**

Forty-six ASD participants were recruited from three special needs schools and 24 normal control were recruited from the community. Among them, 23 age-gender matched ASD and normal control participant-pairs were constructed for the primary analysis. All retinal images were captured using a nonmydriatic fundus camera. Automatic retinal image analysis (ARIA) methodology applying machine-learning technology was used to optimise the information of the retina to develop a classification model for ASD. The model's validity was then assessed using a 10-fold cross-validation approach to assess its validity.

**Findings:**

The sensitivity and specificity were 95.7% (95% CI 76.0%, 99.8%) and 91.3% (95% CI 70.5%, 98.5%) respectively. The area under the ROC curve was 0.974 (95% CI 0.934, 1.000); however, it was noted that the specificity for female participants might not be as high as that for male participants.

**Interpretation:**

Because ARIA is a fully automatic cloud-based algorithm and relies only on retinal images, it can be used as a risk assessment tool for ASD screening. Further diagnosis and confirmation can then be made by professionals, and potential treatment may be provided at a relatively early stage.

**Research in context**

**Evidence before this study**

Autism spectrum disorder (ASD) is a complex neurodevelopmental disorder that begin at a young age and it affects many aspects of the individual's life including social interaction, communication and the way sensory information was being processed. Diagnosis of ASD requires assessment by multi-disciplinary team of professionals mainly relying on questionnaire and requires a lengthy assessment duration. Early intervention is highly recommended in order to achieve a better outcome, however, the lack of an objective screening method especially in young children is a major cause of delayed diagnosis or even misdiagnosis. Previous studies have shown that thinning of retinal nerve fibre layer was significantly associated with high functioning ASD and Asperger Syndrome. It suggested that retinal image may provide critical information for the classification of ASD.

**Added value of this study**

This study has found significant retinal characteristics between ASD and their age-gender-matched control. In particular, ASD subjects have significantly larger optic disc diameter and larger optic cup diameter. In addition, we have shown that it is possible to develop an objective classification model for ASD using machine-learning methods. Retinal images can be obtained from very young children instead of relying solely on lengthy clinical and behavioural assessment. This technique provides an objective screening method that can be implemented in a community setting.

**Implications of all the available evidence**

Risk assessment base on machine-learning for retinal image provides an opportunity for early intervention instead of waiting for lengthy diagnosis. Early intervention has the potential of making a difference in the life of ASD children with long term positive impact. The use of retinal image analysis is noninvasive, fully automatic and relatively convenient.

Alt-text: Unlabelled box

## Introduction

Autism spectrum disorder (ASD) is a complex neurodevelopmental disorder characterised by impairments in social interaction and communication that begin at a young age, which commonly manifest as repetitive behaviours and restricted interests [1]. Diagnosis of ASD requires assessment by a multi-disciplinary team, which often includes a speech and language therapist, a paediatrician, and a psychiatrist or psychologist [2,3,4]. Techniques for detecting signs of disorders usually require a lengthy period of time and are impractical for screening numerous individuals [5,6].

The prevalence of ASD has increased worldwide in recent years. In April 2018, Centers for Disease Control and Prevention released a report regarding prevalence of ASD among children 8 years of age with records from 11 sites of a monitoring network [7]. The overall prevalence of ASD in 2014 was 16.8 per 1000 (i.e., approximately 1 in 59), a significant increase of nearly 15% from 14.7 per 1000 (i.e., approximately 1 in 68) in 2010. The prevalence of ASD ranged from 13.1 to 29.3 per 1000 children aged 8 years in different communities throughout the United States [7]. In a 2017 report released by Focus for Health comparing autism rates across the developed world, Hong Kong was at the top of the list with 37.2 per 1000 (i.e., approximately 1 in 27) [8,9].

ASD diagnosis requires combining information from multi-disciplinary assessments [2,3,4]. The clinical diagnosis is based upon the criteria published in the Diagnostic and Statistical Manual of Mental Disorder – Fourth Edition – Text Revision (DSM-IV-TR; American Psychiatric Association) [10]. By age of 2 years, children can be accurately diagnosed with ASD if they are seen by a highly trained specialist [11,12]. However, the diagnosis is easily being delayed to of age 5 years old. Wiggins et al. (2008) found that there were a variety of diverse factors that may account for the delay of diagnosis, including lack of qualified professionals to make the diagnosis and it is difficult to perform the diagnostic tests in a busy primary care setting [13]. Proper referral after ASD-specific screening can be the key to increase the chance of early identification of ASD children.

Researchers have developed questionnaires to screen for autism under 24 months with follow-up [14,15]. Low sensitivity (ability to identify young children with ASD) and positive predictive value (PPV) are always a concern for screening ASD at young age. Checklist for Autism in Toddlers (CHAT) which is a 2-stage screening tool was developed to identify autism at age 18 months [14]. However, a long-term follow-up study (7–8 years) showed that it only achieved a sensitivity of 0.21 using medium-risk threshold and 0.11 using high risk threshold [14]. PPV for the modified version (M-CHAT) in a mixed sample of low and high-risk sample was 0.59 [16].

The performance of these questionnaires vary widely depending upon the age of the child, severity of symptoms and differences in cut-off scores. The Screening Tool for Autism in Two-Year Olds (STAT) was validated twice by Stone et al. (2004) [17]. They conducted a study with 52 children, using the cut-off score of 2, the STAT had a sensitivity and specificity of 92% and 85%%respectively. In 2008, they conducted another study with 71 high-risk ASD children, using a cut-off score of 2.75, and had a sensitivity of 95% and specificity of 73% [18].

Child psychologists believe that early intervention may help produce better outcome for people with ASD. However, before such intervention can be provided, an early diagnosis is required. Previous studies have shown that ASD can be detected at 14 months of age in some children [19,20] and may achieve high stability for diagnosis by the age of 18 months [21]. However, the lack of a standard screening program for toddlers has led to delays in diagnosis. In the United States, the median age of diagnosis with comprehensive evaluation is 40 months [22]. The situation is similar in Hong Kong, most diagnoses were made between 3 and 4 years of age; hence, the waiting time for a formal diagnosis would be approximately 2 years [23,24]. The same report also identified that some ASD patients with milder autistic features could only be diagnosed after 6 years of age [24]. A suggestion was made to sharpen the awareness of parents and professionals with respect to the early indicators of ASD, as well as the need to develop more sensitive assessment tools for milder ASD cases.

The retina is a sensory membrane that lines the inner surface of the back of the eye. It is an extension of the central nervous system (CNS), and consists of glial cells and retinal ganglion cells and axons, the latter of which form the fibres of the optic nerve. Recent research has used the retinal nerve fibre layer (RNFL) to evaluate associations with brain structural abnormalities. Prospective studies using fundus camera and optical coherence tomography have correlated retinal changes to several CNS disorders such as stroke, multiple scoliosis, Parkinson's disease and Alzheimer's disease [[25], [26], [27], [28]]. In recent years, algorithms for retinal images analysis have also been developed to detect stroke and severity of white matter hyperintensity in the brain [29,30].

Retinal changes are found in autism patients. In a study by Gialloreti et al. (2014)[31] the retinal nerve fibre layer was evaluated in a group of 24 young adults with ASD (11 participants presented with a diagnosis of “high functioning autism” and 13 participants with “Asperger Syndrome”) and 24 participants were healthy controls. Their results provided evidence of retinal variations in different ASDs, with thinner RNFLs found in high-functioning autism individuals, compared with those with Asperger syndrome or healthy controls. Hence, thinning of the RNFL may be a pathophysiological marker of autism. With the discovery of such retinal differences in the autism population, we aimed to develop a screening method by using retinal images for the detection of ASD, and to determine if there are characteristics from fundus images that can distinguish people with ASD from healthy individuals.

## Methods

ASD cases were recruited from the Hong Chi Schools, which is a government-funded schools system for students with ASD and mild to moderate intellectual disabilities. The schools encourage students to participate in activities and training for independence in order to assimilate into society, it also strives to increase the general public's understanding and acceptance of people with intellectual disabilities and ASD. The program for ASD students include counselling training (one-on-one or group of two) and group activities (playgroups, communication group, skills, social, exercise, craft, thinking, voice and sensory).

The control subjects were recruited by an optometrist who has interested in research on children eye care and has experience in taking fundus photo on children. The specific community optometry clinic provides services to school age children from different mainstream schools.

All parents/guardians of children with ASD diagnosis were invited in the three schools. Interested parents/guardians obtained informed consent forms from the class teachers to review the research information at home. Signed informed consent forms were returned to the class teachers if parents agreed their children to participate in the study. A research nurse was in the schools on the day of fundus photography for answering any questions regarding the research and ensuring the written consent was obtained from the parents/guardians before any research procedure was performed. Parents/guardians of children with no ASD diagnosis at the optometry clinic were invited to participate in the research. Written informed consent were also obtained from them before the research procedure.

A total of 70 participants aged between 7 and 20 years were recruited for the study. among them, 46 with a clinical diagnosis of ASD were recruited from three special needs schools for ASD, followed by entering 24 control participants without ASD from a community optometry clinic whose age and gender were matched with those of the ASD participants. After informed consent was obtained from participants’ parents or guardians, retinal images of the participants were taken using a non-mydriatic fundus camera without dilation. A Topcon non-mydriatic retinal camera (TRC-NW400, Tokyo Optical Co, Tokyo) was used to capture a colour retinal image using a 45° field of view centred on the fovea. Participants with eye diseases unsuitable for retinal imaging, such as severe cataract, glaucoma, atretopsia, and corneal plague were excluded. Only participant numbers and initials were obtained to identify retinal images and case report forms during data collection and analysis. The study adheres to STROBE reporting guidelines. The study was conducted in compliance with the Declaration of Helsinki. Ethics approval was obtained from the Joint CUHK-NTEC Clinical Research Ethics Committee.

### Statistical methods

**Fig. 1:**
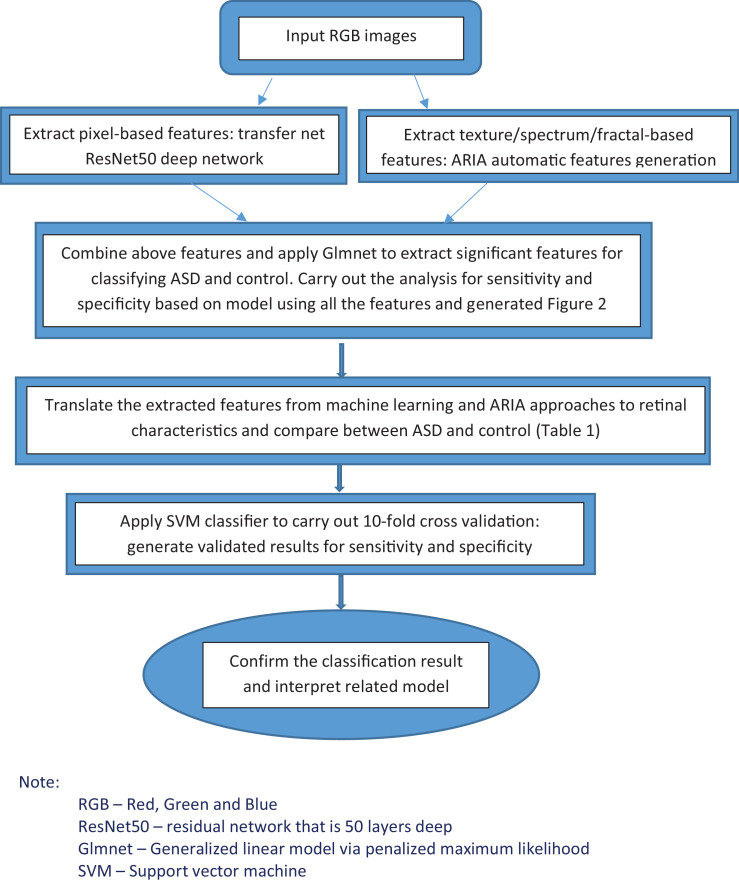
Flowchart of the method presented in our ASD study Note: RGB – Red, Green and Blue ResNet50 – residual network that is 50 layers deep Glmnet – Generalized linear model via penalized maximum likelihood SVM – Support vector machine.(For interpretation of the references to color in this figure legend, the reader is referred to the web version of this article.)

An initial univariate analysis was performed using paired *t*-test to evaluate if the previously known retinal variables were significantly different between the ASD and control groups. Test of normality for the variables were done using Shapiro-Wilk test prior to the use of paired *t*-test. When the test of normality was significant indicated normality assumption was violated, a nonparametric Wilcoxon signed-rank test was used instead. Variables with a p value of less than 0.2 are shown in Table 1; those with p value of less than 0.05 were considered statistically important for further study. For the classification analysis, we used machine learning and deep learning techniques. Using Matlab, we first applied transfer net ResNet-50 convolutional neural network with retinal images (RGB and size 224 × x224 × x3) as input, and the outputs were features generated at the layer of ‘'fc1000_softmax’', based on pixels associated with ASD status [32]. We also extracted the texture/fractal/spectrum related features (such as high order spectra and fractal dimensions) that are associated with ASD by using the automatic retinal image analysis (ARIA) algorithm written in Matlab [33]. We then used the glmnet approach to select the most important subset of features based on the penalised maximum likelihood by using R and Matlab [34,35]. These refined features are highly associated with ASD. Finally, we translated the features extracted from the aforementioned machine learning approaches to commonly used retinal characteristics measured from the images using ImageJ. This part of the analysis helped enhance our understanding of retinal characteristics that contribute to the classification and identification of ASD and was performed with SPSS. We have previously applied this method and validated results in different disease cohorts, including patients with stroke, diabetes, and coronary heart disease [[36], [37], [38], [39]]. For the present study's validation, we applied a 10-fold cross-validation method by using a support vector machine (SVM) algorithm for testing datasets that were not used in the training of the model [34,40]. This was performed by partitioning the dataset and using a subset to train the algorithm, and the remaining subset of data for testing. Each time we ran the cross-validation analysis, we used 10% of the data for testing that were not used at all in the training data. The advantage of this method is that the data used for testing in each run were excluded from the specific training models for the purpose of validation to reduce the problem of overfitting and overestimation of the sensitivity and specificity. Because cross-validation does not use all of the data to build a model, it is a commonly used method to prevent overfitting during training. Fig. 1 shows the flowchart of the described methodology.

**Table 1:** Univariate analysis of retinal characteristics (gender and age-matched).

	Normal (*N* = 23)	ASD (*N* = 23)	*P*-value
Left arterio-venule ratio	.748 (0.722–0.774)	.782 (0.753–0.812)	.062
Left average asymmetry index	.842 (0.818–0.865)	.808 (0.780–0.836)	.090
Left nipping (*probability*)	.326 (0.300–0.353)	.393 (0.362–0.424)	.002
Left hemorrhage (*probability*)	.320 (0.297–0.342)	.356 (0.330–0.381)	.009
Left occlusion (*probability*)	.068 (0.055–0.081)	.080 (0.069–0.092)	.030*
Left exudates (*probability*)	.258 (0.233–0.283)	.308 (0.281–0.336)	.005
Right hemorrhage (*probability*)	.291 (0.275–0.308)	.320 (0.295–0.344)	.051
Right exudates (*probability*)	.225 (0.205–0.246)	.263 (0.232–0.294)	.051
Average cup-to-disc ratio	.414 (0.389–0.438)	.445 (0.413–0.478)	.100
Average disc diameter (µm)	262.0 (250.2–273.9)	279.5 (268.0–290.9)	.013
Average cup diameter (µm)	109.4 (98.9–119.9)	125.5 (112.7–138.4)	.028

Note: The *p*-value with a.

⁎ represents nonparametric test of matched pair data (Wilcoxon Signed-Rank Test), others use paired *t*-test.

### Sample size

For the investigation of individual retinal variables, we noted that Gialloreti et al. (2014) have shown that high-functioning autism (HFA) patients have a reduction of global retinal nerve fibre layer (RNFL) thickness as compared with Asperger Syndrome (AS) patients with 95.0 (SD=8.8) versus 101.9 (SD=10.2) respectively [31]. Participants with AS showed a decreased nasal-quadrant RNFL thickness (71.9, SD=12.0) compared with controls (79.9, SD=6.2). To detect a 10-point difference, with a standard deviation of 12.0 based on study conducted by Gialloreti et al. (2014), using 80% power with a two-sided 5% level test, we need at least 23 participants in each of the ASD and control groups [41]. We therefore recruited ASD participants from three special needs schools until we had 46 eligible participants; then, we recruit healthy controls that matched the ASD participants by age and gender until we achieved the required sample size. In total, we had 23 age and gender matched pairs of ASD and control.

### Role of funding sources

No funding involved in this study.

## Results

The average age of the ASD and control groups were 13.20 and 13.17 years respectively (*p* = 0.852). Furthermore, 36/46 (78.3%) and 17/24 (70.8%) were males participants (*p* = 0.492) in the ASD and control groups, respectively. Because age and gender are considered important risk factors, we matched them both for the analysis. In the univariate analysis of retinal characteristics (Table 1), we have found significantly more nipping (*p* < 0.001), more haemorrhage (*p* = 0.032 and *p* = 0.055 for the left and right eyes, respectively), more exudates (*p* = 0.008 and *p* = 0.042 for left and right eyes, respectively), larger optic disc diameter (*p* = 0.034) and larger optic cup diameter (*p* = 0.050) in the participants with ASD. The univariate analysis revealed that there were differences in retinal characteristics between the ASD participants and their matched normal controls.

**Fig. 2:**
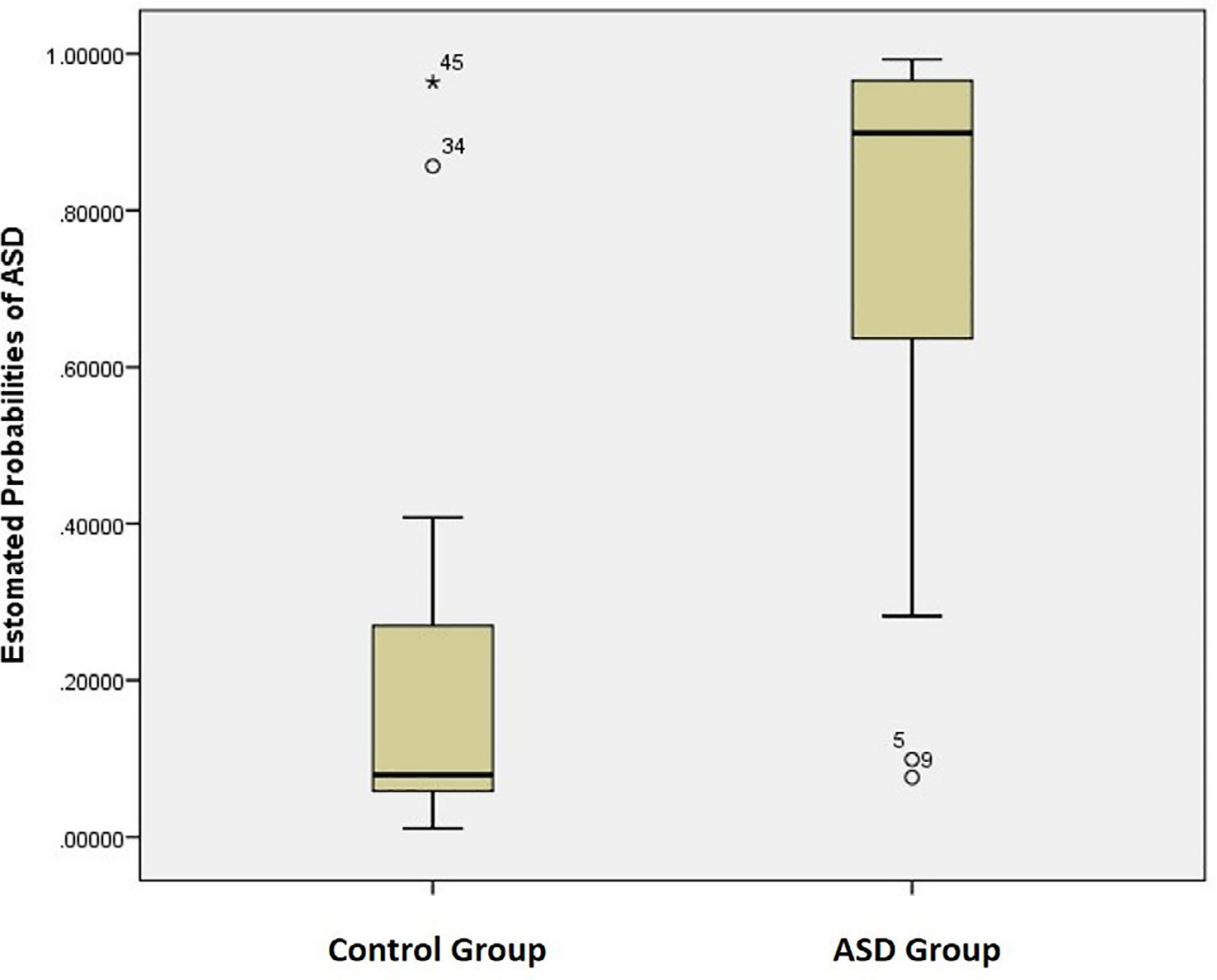
Box plot for the probability of ASD based on the matched case–control data.

After the univariate analysis, we used a generalised linear model based on the penalised maximum likelihood to model additional information for classification. The results of this multivariate model are our primary analysis. Our primary analysis was based on the 23 ASD cases and matched control pairs. Among the 23 matched pairs, 17 (73.9%) pairs were male participants, with an average age of 13.17 years. The sensitivity and specificity for this multivariate classification model were 95.7% (95% CI 76.0%, 99.8%) and 91.3% (95% CI 70.5%, 98.5%) respectively. The area under the ROC curve was 0.974 (95% CI 0.934, 1.000). The probability of ASD was calculated and shown graphically for the cases and controls (Fig. 2). The mean probability of ASD for the controls was 0.205 (95% CI 0.096, 0.314) and the mean probability of ASD for cases was 0.761 (95% CI 0.638, 0.885). We also performed the analysis for all the 70 participants without matching and found that the resulting classification model had a sensitivity of 95.7% (95% CI 84.0%, 99.2%) and specificity of 91.7% (95% CI 71.5%, 98.5%). The area under the ROC curve was 0.983 (95% CI 0.958, 1.000). Overall, the results were highly consistent.

Further investigation on the subgroups was stratified by sex as shown in Table 2. For the male participants, we obtained a sensitivity and specificity of 97.2% (95% CI 83.8%, 99.9%) and 100% (95% CI 77.1%, 100%) respectively. For the female participants, we obtained a sensitivity and specificity of 90.0% (95% CI 54.1%, 99.5%) and 71.4% (95% CI 30.2%, 94.9%) respectively. The specificity for the female participants was significantly lower than that of the male participants as the female specificity of 71.4% was outside of the 95% confidence interval of the male specificity. Although our sample size was relatively small (especially after stratification by gender), the results suggest that because of the lower specificity in the female participants, we are more likely to miss female cases than male cases.

**Table 2:** Subgroup analysis for gender and age.

		N	Sensitivity	95% CI for Sensitivity	Specificity	95% CI for Specificity
Gender	Male	53	97.2%	(83.8%, 99.9%)	100% *	(77.1%, 100%)
	Female	17	90.0%	(54.1%, 99.5%)	71.4% *	(30.2%, 94.9%)
						
Age	<13	24	94.4%	(70.6%, 99.7%)	83.3%	(36.5%, 99.1%)
	>=13	46	96.4%	(79.8%, 99.8%)	94.4%	(70.6%, 99.7%)
Overall		70				

Note:

⁎ represents the comparison between male and female with respect to their specificity, the difference was significant at *p* < 0.05.

When we stratified by age by using the average age of 13 years as a cutoff, we found consistent results. For age less than 13 years, the sensitivity and specificity were 94.4% (95% CI 70.6%, 99.7%) and 83.3% (95% CI 36.5%, 99.1%) respectively. For age greater than or equal to 13 years, the sensitivity and specificity were 96.4% (95% CI 79.8%, 99.8%) and 94.4% (95% CI 70.6%, 99.7%) respectively. There were no significant difference between age groups with respect to both sensitivity and specificity as the 95% confidence intervals of the two age groups completely overlap each other.

### Cross-validation results

To evaluate the validity of the sensitivity and specificity of the overall classification, we performed a 10-fold cross-validation analysis by using an SVM algorithm for testing datasets that were not used in the training of the model. This was performed by 10-fold partitioning of the dataset and repeating the training and testing 10 times. Each analysis used a subset of 90% of the data to train the algorithm and the remaining 10% for testing. This process was repeated 10 times until all 10 folds of the data were tested. We then calculated the overall sensitivity and specificity for the cross-validation analysis based on all 10 independent analyses. The results of the sensitivity and specificity from the cross-validation analysis were 82.6% (95% CI 60.5%, 94.3%) and 91.3% (95% CI 70.5%, 98.5%) respectively. The area under the ROC curve was 0.907 (*p* < 0.001) as shown in Fig. 2.

## Discussion

In our study, we have shown that retinal images can be used to make an objective risk classification for ASD based on various retinal characteristics and nerve fibre-related features as reflected by cup and disc related parameters. By employing machine learning and complex analysis methods, we could detect ASD with much higher accuracy than simply relying on a set of retinal characteristics. The sensitivity and specificity for the classification model were 95.7% (95% CI 76.0%, 99.8%) and 91.3% (95% CI 70.5%, 98.5%) respectively. The area under the ROC curve was 0.974 (95% CI 0.934, 1.000).

Our ASD samples were recruited from the Hong Chi Schools, which is a government-funded schools system for students with ASD and mild to moderate intellectual disabilities. The ASD students were formally diagnosed as they received government funding and represent children from general public and not only for privileged high income class in the society. In addition Hong Kong has adopted DSM-IV as the criteria for diagnosis of ASD, which are used by many countries [42]. The organization highly supports teaching staff to participate in professional exchange and consultation projects with the Mainland and overseas partners to increase their professional knowledge and are up-to-date with international community. With the advantages of the sampling of ASD cases and the age and gender matched control from the local community, we would have more confidence that our findings may generalize to a wider population of children with autism spectrum disorder.

Autism is becoming a public health issue as the number of reported cases has been increasing. Muhle et al. (2004) stated that 556% increase in pediatric prevalence between 1991 and 1997 in US.[1] The prevalence was considered higher than that of spina bifida, cancer, or Down syndrome. US Centers for Disease Control and Prevention revealed that one in 68 children now have a diagnosis of ASD which is a 30 percent increase in just 2 years. In 2002, about one in 150 children was considered autistic and in 1991 the figure was one in 500 [43].

Autism is not a disease but a neurodevelopment disorder which characterized by impairment of social and behavioural domains. To optimize long-term prognosis, early identification of children at risk for autism is critical because these are critical times for early social and language development at age of 18 and 24 months. A study revealed that children who began treatment between 18 and 23 months of age improved significantly more than the other three groups (24–29 months, 30–36 months, 37–48 months) [44]. In addition, early identification could also help parents to prepare for their family financial planning. Dudley and Emery (2014) has estimated the financial support to live with an ASD individual [45]. If a child is severely impacted and requires constant and lifelong supports, then the value of caregiver time required to support that individual is approximately $5.5 million higher than that for someone without autism. The cost of saving to the society by saving one ASD child is high, and current evidence is that starting intervention as early as 6 months or 1 year may increase the chance of saving an ASD.

Previous studies have found differences in brain characteristics between ASD and typical children. In young children with ASD around 2–4 years of age, the total brain volume growth is quicker [46,47]. The condition seems to be predominated by the enlarged volume of the frontal and temporal lobes, whose accelerated growth stops after around 10–15 years of age [48,49]. A recent study has reported that this early overgrowth of the brain is caused by an accelerated expansion of the cortical surface area, but not cortical thickness, before the age of 2 years [50]. This accelerated expansion of the cortical surface area of the grey matter in ASD also appears to be associated with impaired maturation of the cortical white matter. These early white matter differences in ASD brains might explain the brain being connected atypically [51].

Lange et al. (2010) used MRI to measure brain circuity deviations in a group of 60 participants (30 ASD and 30 controls) [52]. They studied the white matter microstructure (WMM) in the superior temporal gyrus (STG) and temporal stem (TS) which contains circuitry central to language, emotion, and social cognition. They found that in ASD cases, tensor skewness was greater on the right and fractional anisotropy was decreased on the left. They also found increased diffusion parallel to white matter fibres bilaterally. The increased white matter cerebral blood flow, or increased role of altered maturation in white matter, could be factors leading to the decrease of structural and functional connectivity in the human brain [53,54]. These phenomena may explain symptoms exhibited by people with ASD. Although MRI is a valuable tool for advancing our understanding of cerebrovascular pathophysiology, it is invasive (in a sense that the child has to be placed in a noisy, confined space alone for quite some time), time–consuming, expensive, and available only in specialised centres. To provide widespread screening of people at risk of autism, a simpler, more accessible technique is required. Our method may be used as a community-based risk assessment tool instead of a diagnostic tool to offer parents the opportunity to provide early intervention to children with high risk of ASD.

However, we must also recognised a few limitations of our study. First our sample size is relatively small; although we matched age and gender to increase statistical power and control for potential confounding effects, we cannot eliminate the possibility that other potential confounders, such as concomitant diseases or comorbidities, may have an effect. Second, we do not have a lot of clinical information for the participants as the recruitment was carried out in school setting instead of clinical setting. Third, it may be useful to obtain family background and information that may be used to assess outcome in long-term follow-up. Lastly, it would add more credibility for the scientific investigation if we have magnetic resonance imaging (MRI) in order to enhance the interpretation of the retinal image analysis.

## Declaration of Competing Interest

BZ and JL have a patent “Method and device for retinal image analysis” licensed to Health View Bioanalytic Limited, received royalties through the Chinese University of Hong Kong. BZ and JL are founders and shareholders of Health View Bioanalytic Limited, Bioanalytic Holdings Limited, and Bioanalytic International Holdings Limited. ML is director of Bioanalytic Holdings Limited, and Bioanalytic International Holdings Limited. BZ is co-founder and shareholder of Beth Bioinformatics Company Limited. ML reports Spouse of Founder and Shareholder of Health View Bioanlaytic Limited; Honorary Consultant of Health View Bioanalytic Limited. The remaining authors have no conflict of interest to declare.

## Appendix Supplementary materials

Image, application 1
